# Supplementary material for: Leukocyte immunoglobulin-like receptor B1 (LILRB1) protects human multiple myeloma cells from ferroptosis by maintaining cholesterol homeostasis
Source: Nat Commun. 2024 Jul 9;15:5767. doi: 10.1038/s41467-024-50073-x (PMC11233649; doi:10.1038/s41467-024-50073-x)
Supplement: Supplementary file 1 — Supplementary Information [file 41467_2024_50073_MOESM1_ESM.docx]

**Supplementary figures**

**
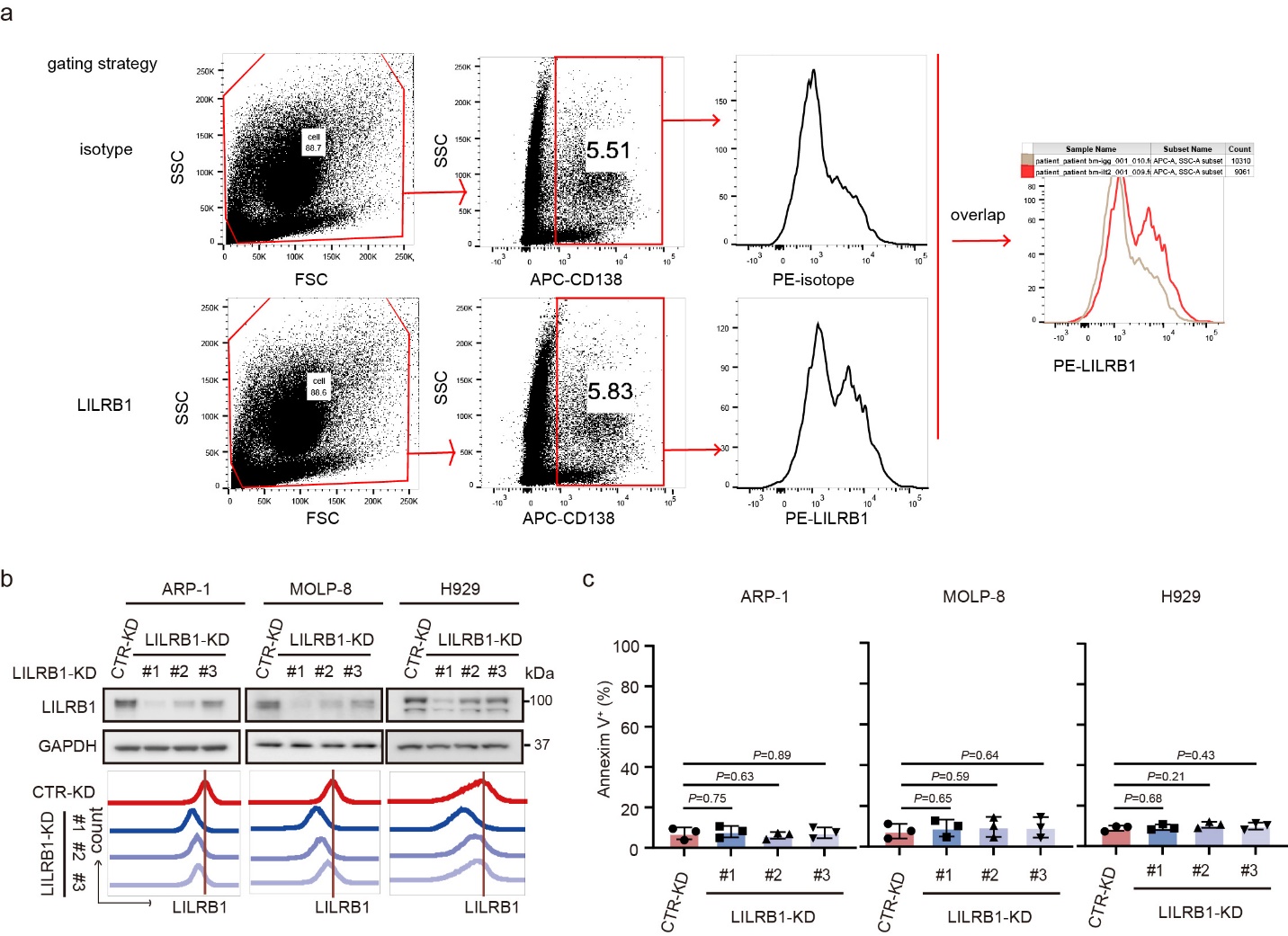
**

**Supplementary Fig. 1. Effect of LILRB1 KD in MM cells in vitro.**

**(a)** Gating strategy of Fig. 1j. **(b)** KD efficiency of LILRB1 by three different shRNAs (#1, #2 and #3) in ARP-1, MOLP-8, and H929 cells determined by western blot and flow cytometry. The independent experiments were repeated three times and the representative images are shown. **(c)** Apoptosis of CTR-KD and LILRB1-KD MM cells detected by flow cytometry. n = 3, independent experimental repeats. Statistical significance was determined by two-tailed Student t-test. Source data are provided as a Source Data file.


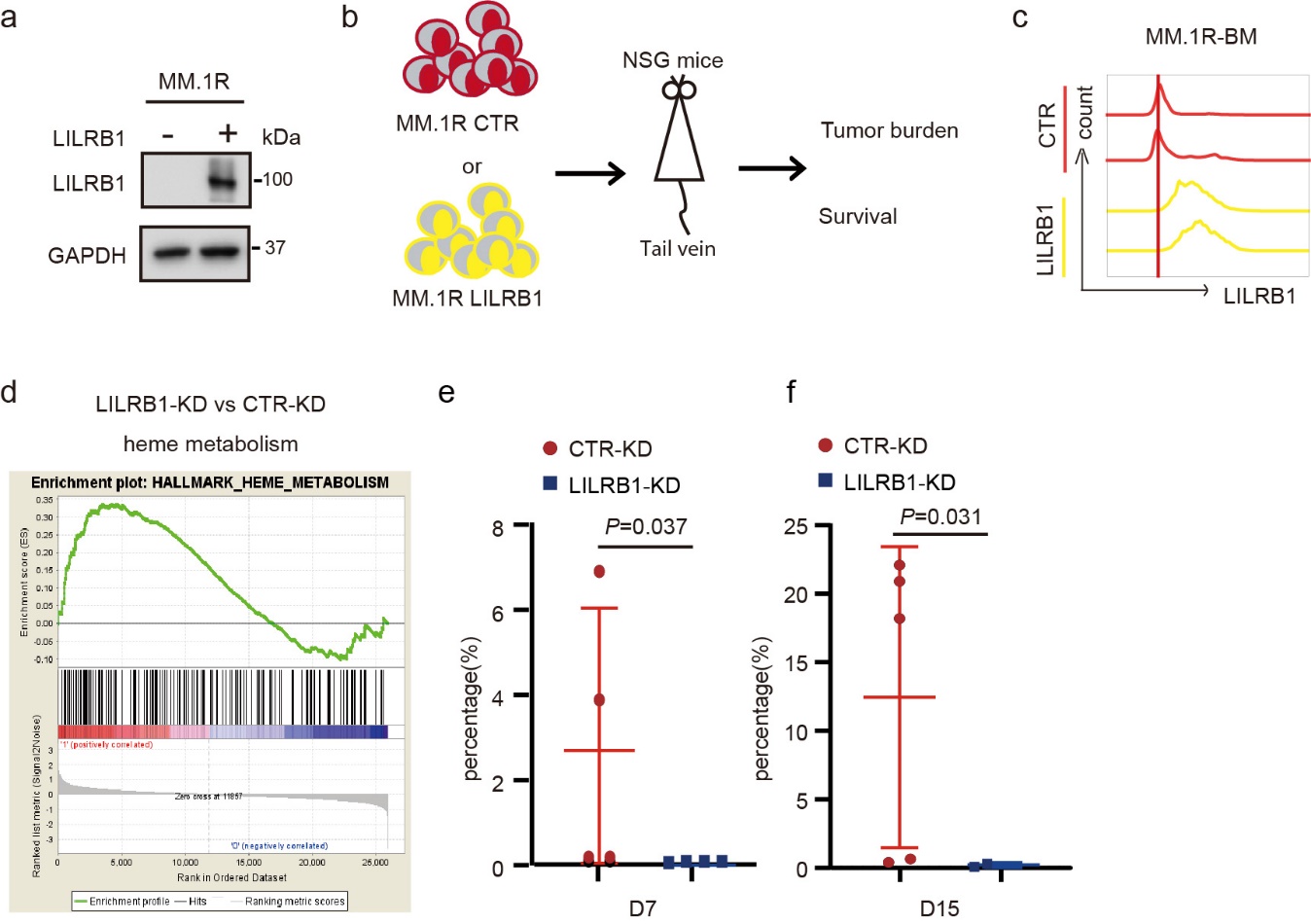


**Supplementary Fig. 2. Function of LILRB1 on MM development in vivo**

**(a)** Overexpression of LILRB1 in MM.1R cells confirmed by western blot. The independent experiments were repeated three times and the representative images are shown. **(b)** Schematic presentation of experimental procedure. NSG mice were injected with 2 × 10^6^ CTR- or LILRB1-overexpressing MM.1R cells, followed by monitoring of tumor burden and survival. **(c)** LILRB1 expression in MM.1R cells from the BM of CTR- or LILRB1-overexpressing MM.1R-bearing mice to confirm the overexpression of LILRB1. **(d)** Gene set enrichment analysis of heme metabolism of RNAseq data of CTR-KD and LILRB1-KD ARP-1 cells sorted from the bone marrow of CTR-KD or LILRB1-KD ARP-1-bearing NSG mice. **(e-f)** NSG mice were injected with 5 × 10^6^ CTR-KD or LILRB1-KD ARP-1 cells. After 7(e, n=4) or 15 (f, n=5) days of injection, the percentage of MM cells in the BM was detected. n, biological repeats, different mice samples. Statistical significance was determined by two-tailed Student t-test. Source data are provided as a Source Data file.


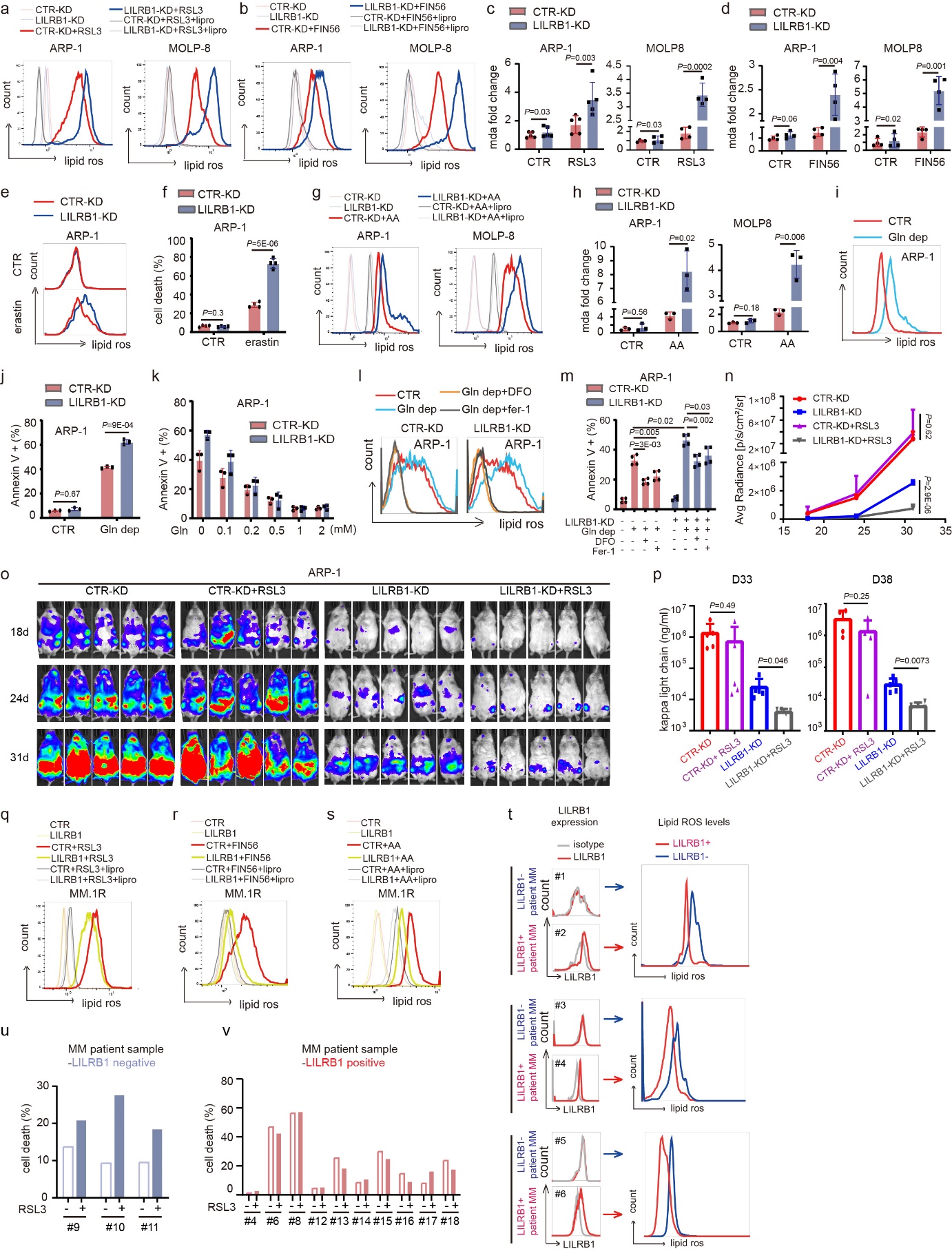


**Supplementary Fig. 3. LILRB1 deficiency enhances MM cell susceptibility to ferroptosis. (a,b,e,g)** CTR-KD or LILRB1-KD MM cells were treated with ferroptosis inducer RSL3 (400 nM) **(a)**/FIN56 (15 μM) **(b)**/erastin (5 μM) **(e)**/ AA (75 μM) **(g)** and Liproxstatin-1 (lipro, 1 μM). Lipid peroxidation was measured, and the representative histograms are shown. **(c,d,h)** CTR-KD or LILRB1-KD MM cells were treated with ferroptosis inducer RSL3 (400 nM) **(c**, ARP-1 n=5, MOLP-8 n=4**)**, FIN56 (15 μM) **(d,** n=4**)**, or AA (75 μM) **(h,** n=3**)** and lipid peroxidation was measured by malondialdehyde (MDA) assay. **(f,** n=4**)** CTR-KD or LILRB1-KD ARP-1 cells were treated with erastin (5 μM) and cell death was measured. **(i-j)** ARP-1 cells were cultured in normal or glutamine (Gln)-deprived medium and lipid peroxidation **(i)** and cell death were measured **(j**, n=3**)**. **(k**, n=3**)** CTR-KD or LILRB1-KD ARP-1 cells were cultured in medium with addition of different concentrations of glutamine and cell death was determined. **(l,m)** CTR-KD or LILRB1-KD ARP-1 cells were cultured with glutamine-deprived medium in the presence of ferroptosis inhibitor deferoxamine (DFO) or ferrostatin-1 (Fer-1), followed by measurement of lipid peroxidation **(l)**, and cell death determined by annexin V staining **(m**, n=4**)**. **(n-p)** NSG mice were injected with 2 × 10^6^ CTR-KD or LILRB1-KD ARP-1 cells, followed by administration of vehicle or RSL3 (20 mg/kg, ip) biweekly and monitoring of tumor burden (n=5). Representative bioluminescent imaging for tumor burden **(o)** and summarized quantification of bioluminescent imaging (mean ± SD) **(n)** are shown. **(p)** Tumor burden was measured as serum concentration of κ light chain and shown as mean ± SD. **(q-s)** CTR- or LILRB1-overexpression MM.1R cells were treated with ferroptosis inducer RSL3 (400 nM) **(q)**/FIN56 (15 μM) **(r)**/AA (75 μM) **(s)** and Liproxstatin-1 (lipro, 1 μM). Lipid peroxidation was measured, and the representative histograms are shown. **(t)** Lipid peroxidation of primary MM cells with negative/positive expression of LILRB1 was measured. **(u,v)** Primary MM cells with negative (LILRB1^-^) **(u)** / positive (LILRB1^+^) expression **(v)** of LILRB1 were treated with RSL3 and cell death was detected. For **(c,d,f,h,j,k,m,p)**, data are presented as mean ± SD. For **(c,d,f,h,j,k,m)**, n, independent experimental repeats. For **(n-p)**, n, biological repeats, different mice samples. Statistical significance was determined by two-tailed Student t test. Source data are provided as a Source Data file.


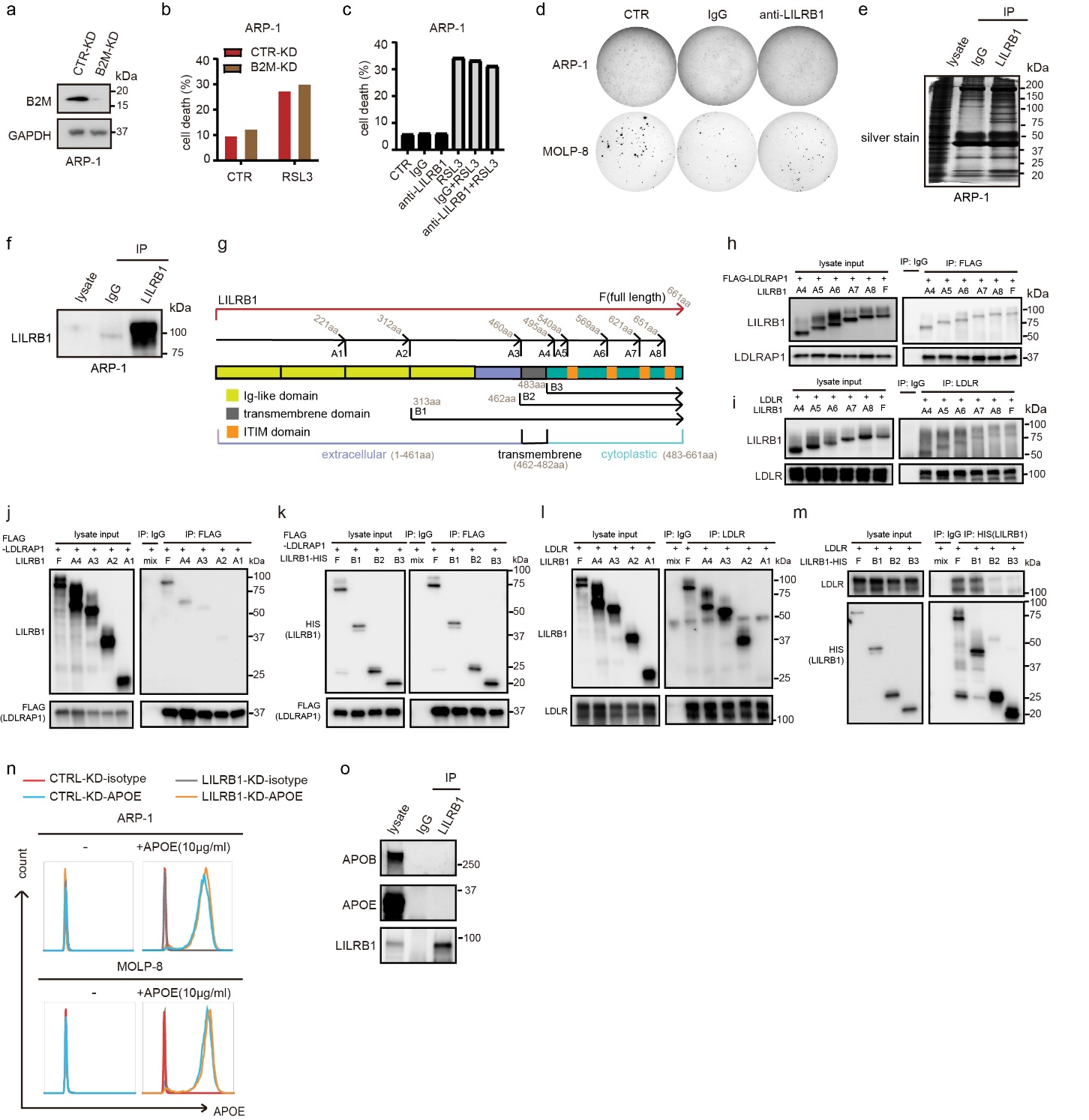


**Supplementary Fig. 4. LILRB1 plays an important role in LDL uptake.**

**(a)** KD efficiency of B2M in ARP-1 confirmed by western blot. **(b)** Cell death of CTR-KD or B2M-KD MM cells detected after 10-hour incubation with RSL3. **(c)** ARP-1 cells incubated without or with anti-IgG or anti-LILRB1 antibody (5 μg/ml) for 2 hours and then treated with RSL3. Cell death of CTR-KD or B2M-KD MM cells was detected after 10-hour incubation with RSL3. **(d)** MM cells were planted in soft agar culture medium without or with anti-IgG or anti-LILRB1 antibody (5 μg/ml). Soft agar colony-formation ability was examined 2-3 weeks later by imaging. **(e,f)** Quality control of mass spectrum sample: **(e)** Silver stain showing the proteins in the product of immunoprecipitation by anti-IgG or anti-LILRB1 antibody in ARP-1 cell lysates. **(f)** Western blot showing the expression of LILRB1 in the pull-down product of anti-IgG or anti-LILRB1 antibody. **(g)** Schematic diagram of the structure of LILRB1 and the design of different truncated LILRB1 plasmids. **(h-m)** immunoprecipitation showing the interaction between LDLR/LDLRAP1 and different truncated LILRB1 in 293T cells overexpressed with LDLR/LDLRAP1 and truncated LILRB1. **(n)** CTR-KD or LILRB1-KD MM cells incubated without or with APOE (10 μg/ml) for one hour. After incubation, cells were washed with PBS. Flow cytometry was used to detect the expression of APOE on the surface of MM cells. **(o)** Immunoprecipitation with anti-LILRB1 antibody was performed to detect the interaction between LILRB1 and APOE/APOB in ARP-1 cell lysates. For **(a-d)** and **(h-o)**, the independent experiments were repeated three times, and the representative data are shown. Source data are provided as a Source Data file.


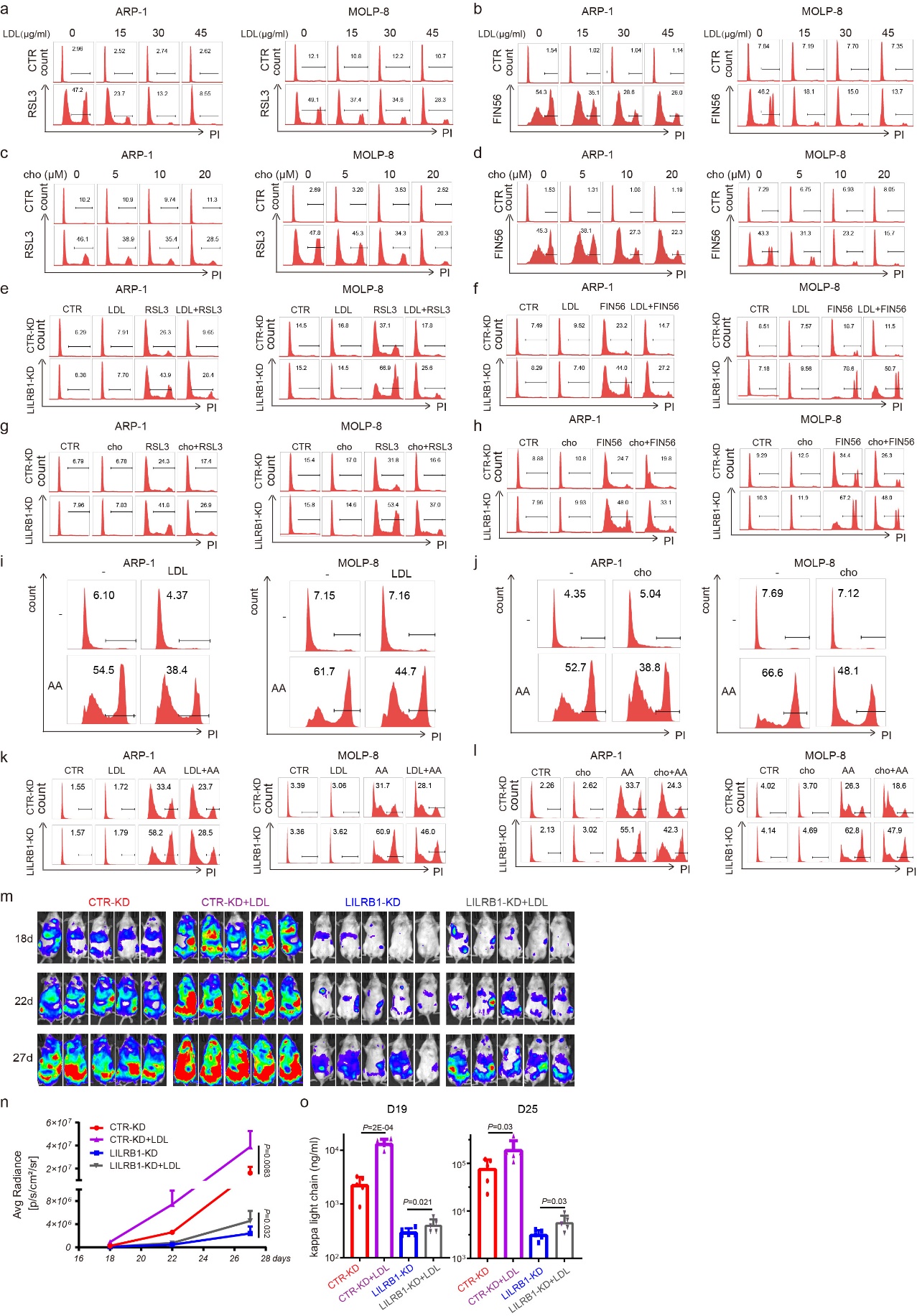
**Supplementary Fig. 5. Cholesterol/LDL protects MM cells from ferroptotic cell death**

**(a-l)** Representative histograms showing cell death of ARP-1 and MOLP-8 MM cells (referred to summarized data in Fig. 5a-l). Cells were incubated with LDL/cholesterol for 12 hours before addition of ferroptosis inducers. Cell death detected after 10-hour incubation with LDL or RSL3 (400 nm) alone or their combination **(a)**, and cholesterol or RSL3 alone or their combination **(c)**; or after 24-hour incubation with LDL or FIN56 (15 µM) alone or their combination **(b),** and cholesterol or FIN56 alone or their combination **(d)**. Representative histograms showing cell death of CTR-KD or LILRB1-KD ARP-1 and MOLP-8 MM cells detected after 10-hour incubation with LDL or RSL3 (400 nm) alone or their combination **(e),** and cholesterol or RSL3 alone or their combination **(g)**; and after 24-hour incubation with LDL or FIN56 (15 µM) alone or their combination **(f),** and cholesterol or FIN56 alone or their combination **(h)**. Cell death of MM cells after 10-hour incubation with LDL or AA (75 µm) alone or their combination **(i),** and cholesterol or AA alone or their combination **(j)**, and cell death of CTR-KD or LILRB1-KD MM cells after 10-hour incubation with LDL or AA (75 µM) alone or their combination **(k),** and cholesterol or AA alone or their combination **(l****)**. **(m-o)** NSG mice were injected with 2 × 10^6^ CTR-KD or LILRB1-KD ARP-1 cells with/ or without LDL (10 mg/kg, iv) through tail vein, followed by administration of vehicle (veh) or LDL (10 mg/kg, iv) biweekly and monitoring of tumor burden (n=5). Representative bioluminescent imaging for tumor burden **(m)** and summarized quantification of bioluminescent imaging (mean ± SD) **(n)** are shown. **(o)** Tumor burden was measured as serum concentration of κ light chain. Data are presented as mean ± SD. For **(m-o)**, n, biological repeats, different mice samples. Statistical significance was determined by two-tailed Student t test. Source data are provided as a Source Data file.

**
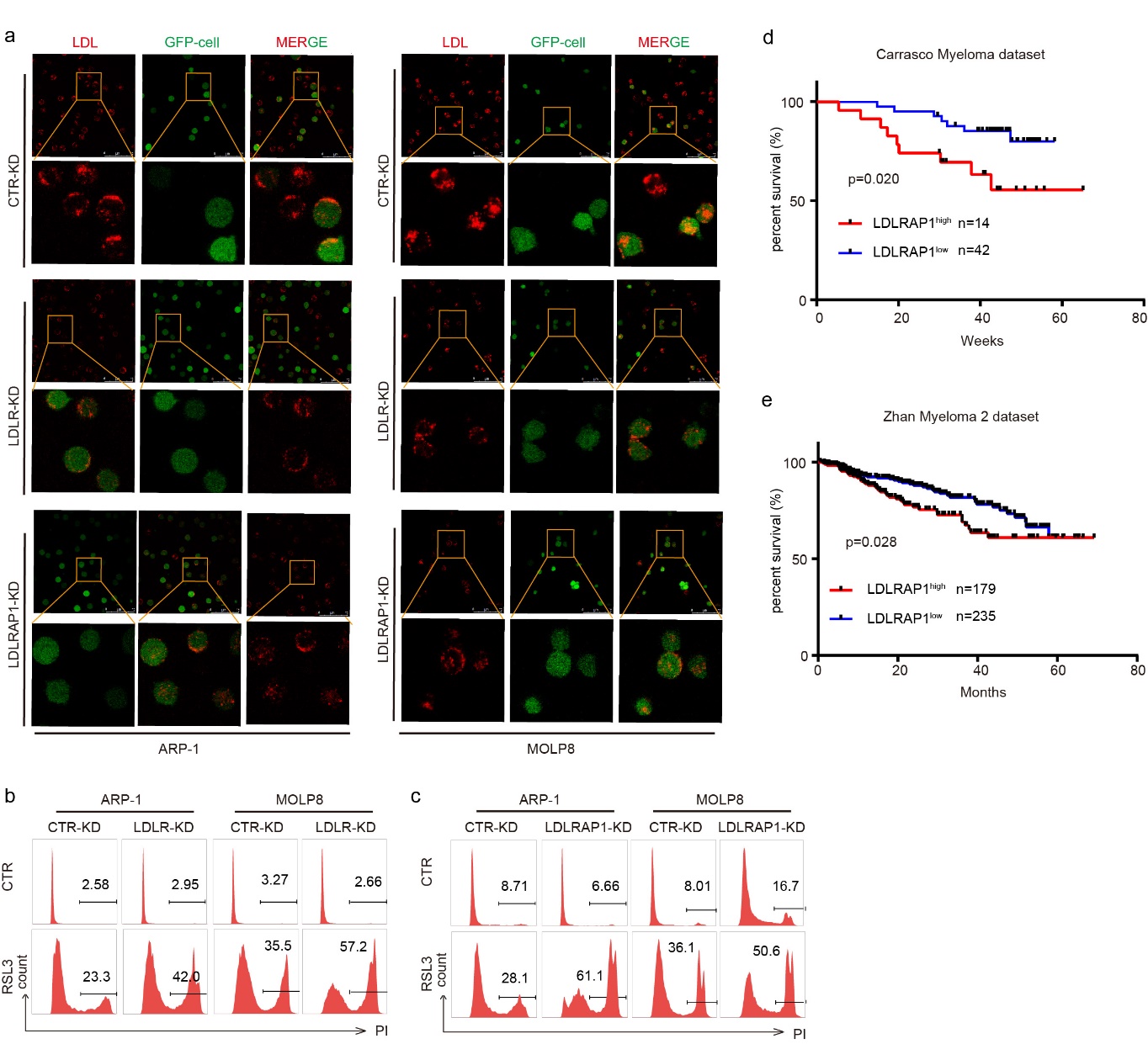
**

**Supplementary Fig. 6. Role of LDLR and LDLRAP1 in LDL uptake**

**(a)** CTR-KD, LDLR-KD, or LDLRAP1-KD MM cells cultured in FBS-free medium for 24 hours and then incubated with chemically modified LDL labelled with red fluorescence for 2 hours. Representative fluorescent confocal images showing LDL uptake by MM cells. Independent experiments were repeated three times, and the representative data are shown. **(b)** Cell death induced by RSL3 (400 nm, 10 hours) in CTR-KD or LDLR-KD MM cells was detected by PI staining with flow cytometry and the representative histograms were shown (referred to summarized data in Fig. 6g). **(c)** Cell death induced by RSL3 (400 nm, 10 hours) in CTR-KD or LDLRAP1-KD MM cells was detected by PI staining with flow cytometry and the representative histograms were shown (referred to summarized data in Fig. 6h). **(d,e)** Survival of MM patients with high LDLRAP1 (LDLRAP1^high^) and low LDLRAP1 (LDLRAP1^low^) expression in Carrasco’s MM dataset **(d)** and Zhan’s MM 2 dataset **(e)**. Statistical significance was determined by Log-rank (Mantel-Cox) test and p values are shown. Source data are provided as a Source Data file.

**
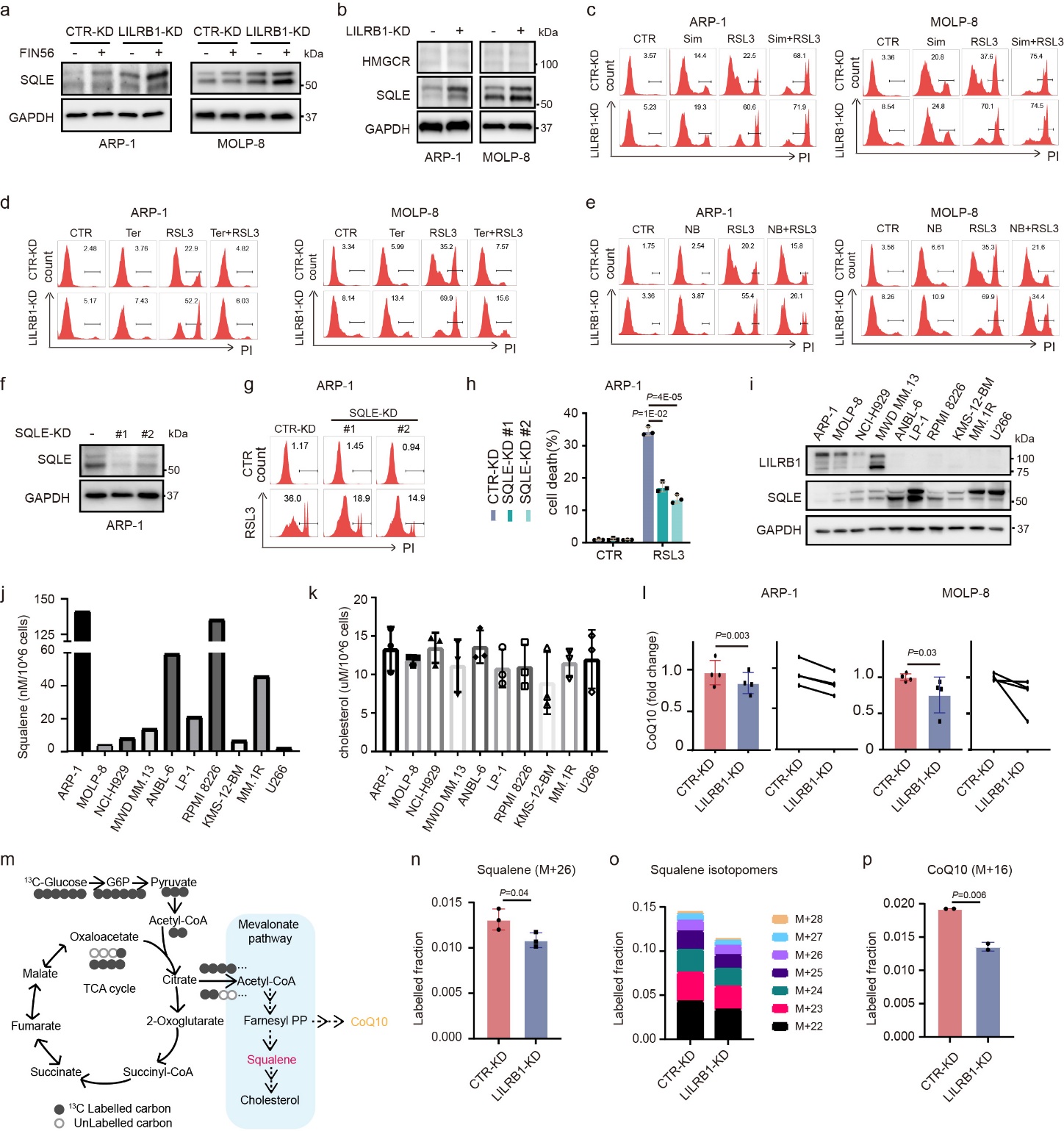
**

**Supplementary Fig. 7. Cholesterol metabolic alteration renders LILRB1-deficient MM cells sensitivity to ferroptosis by upregulating SQLE and downregulating squalene**

**(a)** Western blot showing the expression of SQLE and GAPDH in CTR-KD and LILRB1-KD MM cells treated without or with FIN56. **(b)** Western blot showing the expression of HMGCR, SQLE, and GAPDH in CTR-KD and LILRB1-KD MM cells. **(c-e)** Representative histograms showing cell death of CTR-KD and LILRB1-KD MM cells after incubation without or with RSL3 or simvastatin (Sim) alone or their combination **(c)**, RSL3 or terbinafine HCl (Ter) alone or their combination **(d)**, or RSL3 or NB598 (NB) alone or their combination **(e)** (referred to summarized data in Fig. 7d-f). **(f)** Western blot confirming the knock down efficiency of SQLE. **(g,h)** Cell death induced by RSL3 (400 nm, 10 hours) in CTR-KD or SQLE-KD MM cells was detected by PI staining with flow cytometry. Both the representative histograms **(g)** and summarized data **(h)** are shown. n=3, independent experimental repeats. **(i)** Western blot showing the expression of LILRB1, SQLE, and GAPDH among different MM cell lines. **(j)** Squalene levels among different MM cell lines by HPLC-MS. **(k)** Intracellular cholesterol levels among different MM cell lines by cholesterol assay kit (STA-390, CELL BIOLABS, INC). n=3, independent experimental repeats. **(l)** CoQ10 levels in CTR-KD and LILRB1-KD MM cells were detected by ELISA. n=4, independent experimental repeats. **(m)** Schematic presentation of analysis of [^13^C]glucose uptake to trace incorporation of ^13^C into newly synthesized squalene and CoQ10. **(n-p)** CTR-KD and LILRB1-KD ARP-1 cells were cultured in [^13^C]glucose-containing medium for 24 h and harvested for isotope tracing analysis. **(n)** The labeled fractions of Squalene (M+26) are shown. n=3, biological repeats, independent experimental samples. **(o)** The labeled fractions of Squalene isotopomers (M+22, …, M+28) are shown. **(p)** The labeled fractions of CoQ10 (M+16) are shown. n=2, biological repeats, independent experimental samples. For **(a,b,f,i)**, independent experiments were repeated three times and the representative images are shown. For **(h,k,n,l,p)**, data are shown as mean ± SD. Statistical significance was determined by two-tailed Student t test. Source data are provided as a Source Data file.


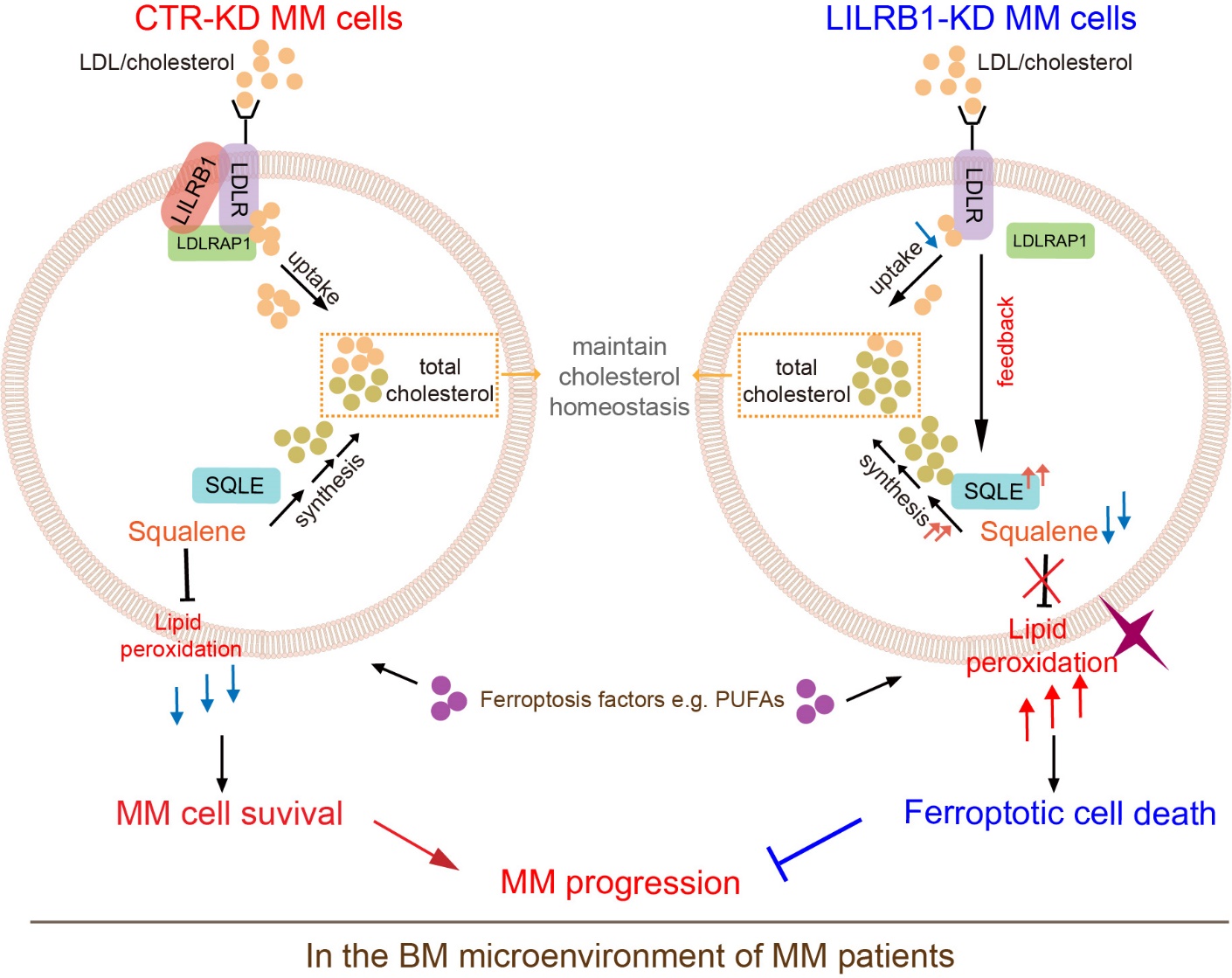


**Supplementary Fig. 8. Schematic model of LILRB1’s function in MM progression**

In the bone marrow (BM) microenvironment of MM patients, ferroptosis-inducing factors such as PUFAs induce lipid peroxidation in MM cells. Normally, LILRB1 expressing on MM cells interacts with LDLRAP1 and LDLR, facilitating LDL/cholesterol uptake and maintaining cell harmony. At this status, the basal squalene level protects MM cells from lipid peroxidation accumulation, preventing ferroptosis and promoting cell survival and MM progression. However, in LILRB1-KD cells, the interaction between LDLR and LDLRAP1 is inhibited, leading to reduced LDL uptake. To maintain cholesterol homeostasis, compensatory cholesterol synthesis is triggered in LILRB1-KD cells by upregulating the expression of cholesterol synthesis rate‐limiting enzyme SQLE, which converts squalene, an anti-ferroptotic metabolite, to (S)-2,3-epoxysqualene. With decreased squalene levels, accumulation of lipid peroxidation in LILRB1-KD MM cells makes them more susceptible to ferroptotic cell death, thus inhibiting MM progression.


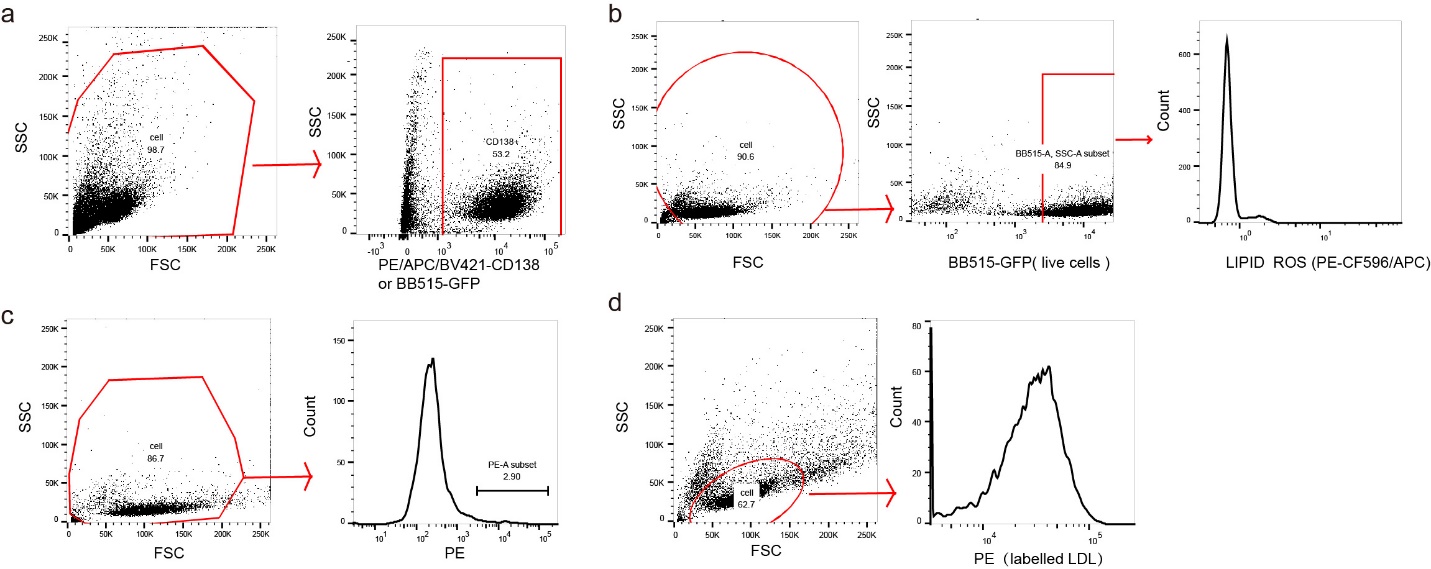


**Supplementary Fig. 9. Gating strategies for flow cytometry (a)** gating strategy of human MM cells in the murine bone marrow for Fig. 2g,h,l,r,s. **(b)** gating strategy of human MM cell lines to detect lipid ROS for Fig. 3a,b,e,j,l. **(c)** gating strategy of human MM cell lines to detect the percentage of cell death for Fig. 3c,d,f,k,m, Fig. 5a-l, Fig. 6g,h, Fig. 7d-f. **(d)** gating strategy of human MM cell lines to detect the uptake of labelled LDL for Fig. 4k, Fig. 6e,f.
