# Supplementary material for: Leukocyte immunoglobulin-like receptor B1 (LILRB1) protects human multiple myeloma cells from ferroptosis by maintaining cholesterol homeostasis
Source: Nat Commun. 2024 Jul 9;15:5767. doi: 10.1038/s41467-024-50073-x (PMC11233649; doi:10.1038/s41467-024-50073-x)
Supplement: Supplementary file 3 — Description of Additional Supplementary Information [file 41467_2024_50073_MOESM3_ESM.docx]

Inventory of Supporting Information

1. Supplementary figures: Supplementary figure. 1-9 (Source data are provided as a Source Data file)

2. Supplementary data 1. genes with significant different expression between MM patients with poor survival and MM patients with good survival

3. Supplementary data 2. MS analysis showed the potential interacting proteins of LILRB1
