# Supplementary material for: Leukocyte immunoglobulin-like receptor B1 (LILRB1) protects human multiple myeloma cells from ferroptosis by maintaining cholesterol homeostasis
Source: Nat Commun. 2024 Jul 9;15:5767. doi: 10.1038/s41467-024-50073-x (PMC11233649; doi:10.1038/s41467-024-50073-x)
Supplement: Supplementary file 6 — Reporting Summary [file 41467_2024_50073_MOESM6_ESM.pdf]

nature portfolio

Corresponding author(s): Qing Yi

Last updated by author(s): May 28, 2024

Reporting Summary

Nature Portfolio wishes to improve the reproducibility of the work that we publish. This form provides structure for consistency and transparency in reporting. For further information on Nature Portfolio policies, see our Editorial Policies and the Editorial Policy Checklist.

Statistics

For all statistical analyses, confirm that the following items are present in the figure legend, table legend, main text, or Methods section.

n/a	Confirmed	
☐	☒	The exact sample size (*n*) for each experimental group/condition, given as a discrete number and unit of measurement
☐	☒	A statement on whether measurements were taken from distinct samples or whether the same sample was measured repeatedly
☐	☒	The statistical test(s) used AND whether they are one- or two-sided<br>*Only common tests should be described solely by name; describe more complex techniques in the Methods section.*
☒	☐	A description of all covariates tested
☒	☐	A description of any assumptions or corrections, such as tests of normality and adjustment for multiple comparisons
☐	☒	A full description of the statistical parameters including central tendency (e.g. means) or other basic estimates (e.g. regression coefficient) AND variation (e.g. standard deviation) or associated estimates of uncertainty (e.g. confidence intervals)
☐	☒	For null hypothesis testing, the test statistic (e.g. *F*, *t*, *r*) with confidence intervals, effect sizes, degrees of freedom and *P* value noted<br>*Give P values as exact values whenever suitable.*
☒	☐	For Bayesian analysis, information on the choice of priors and Markov chain Monte Carlo settings
☒	☐	For hierarchical and complex designs, identification of the appropriate level for tests and full reporting of outcomes
☒	☐	Estimates of effect sizes (e.g. Cohen's *d*, Pearson's *r*), indicating how they were calculated

Our web collection on statistics for biologists contains articles on many of the points above.

Software and code

Policy information about availability of computer code

Data collection	BD FACSDiva software8.1
Data analysis	GraphpadPrismv7.04,FlowJo10.4,GSEAv4.0.3, Trimmomatic/0.38, Star/2.7.10a, R/4.2.9, edgeR, Deseq2, IPA

For manuscripts utilizing custom algorithms or software that are central to the research but not yet described in published literature, software must be made available to editors and reviewers. We strongly encourage code deposition in a community repository (e.g. GitHub). See the Nature Portfolio guidelines for submitting code & software for further information.

Data

Policy information about availability of data

All manuscripts must include a data availability statement. This statement should provide the following information, where applicable:

- Accession codes, unique identifiers, or web links for publicly available datasets
- A description of any restrictions on data availability
- For clinical datasets or third party data, please ensure that the statement adheres to our policy

All raw sequencing data generated in this study have been deposited in the NCBI Gene Expression Omnibus (GEO) under accession number GSE226821 (https://www.ncbi.nlm.nih.gov/geo/query/acc.cgi?acc=GSE226821). The mass spectrometry proteomics data (Mass spectrum analysis to identify the interacting protein of LILRB1 in multiple myeloma cell line ARP-1) have been deposited to the ProteomeXchange Consortium via the PRIDE91 partner repository with the dataset identifier PXD045817 (http://www.ebi.ac.uk/pride/archive/projects/PXD045817).

Gene sets for GSEA analysis including hallmark gene sets and ontology gene sets, are available from The Molecular Signature Database (MSiDB). Canonical Pathway Analysis in IPA is commercially available in QIAGEN (https://digitalinsights.qiagen.com/products-overview/discovery-insights-portfolio/analysis-and-visualization/qiagen-ipa/).
Other previous published MM patient datasets used in the study can get access with the following accession number and link: GSE2658(https://www.ncbi.nlm.nih.gov/geo/query/acc.cgi?acc=GSE2658); GSE4452(https://www.ncbi.nlm.nih.gov/geo/query/acc.cgi?acc=GSE4452); GSE5900 (https://www.ncbi.nlm.nih.gov/geo/query/acc.cgi?acc=GSE5900); GSE19784(http://www.ncbi.nlm.nih.gov/geo/query/acc.cgi?acc=GSE19784); phs000748(https://www.ncbi.nlm.nih.gov/projects/gap/cgi-bin/study.cgi?study_id=phs000748.v7.p4).
All the other data supporting the findings of this study are available with in the article,supplementary information, source files.

Research involving human participants, their data, or biological material

Policy information about studies with human participants or human data. See also policy information about sex, gender (identity/presentation), and sexual orientation and race, ethnicity and racism.

Reporting on sex and gender	no sex or gender information was Involved in our experiment.
Reporting on race, ethnicity, or other socially relevant groupings	These information was not involed.
Population characteristics	These information was not involed.
Recruitment	We have no access to the information of the MM patients due to the policy of privacy protection of patients. When MM patients come to our hospital, we got the chance to get their BM aspirates if they signed the informed consent and allowed us to use their samples.
Ethics oversight	To get primary multiple myeloma cells, BM aspirates were obtained from patients newly diagnosed with MM. This study was approved by the institutional review board at the Houston Methodist Research Institute. Written informed consent was obtained from all patients.

Note that full information on the approval of the study protocol must also be provided in the manuscript.

Field-specific reporting

Please select the one below that is the best fit for your research. If you are not sure, read the appropriate sections before making your selection.

[x] Life sciences [ ] Behavioural & social sciences [ ] Ecological, evolutionary & environmental sciences

For a reference copy of the document with all sections, see nature.com/documents/nr-reporting-summary-flat.pdf

Life sciences study design

All studies must disclose on these points even when the disclosure is negative.

Sample size	Sample size was determined to be adequate based on the magnitude and consistency of measurable differences between groups.
Data exclusions	No data were excluded.
Replication	Replication was verified in individual biological replicates and studies as detailed in each figure legend.
Randomization	For in vivo mouse experiments,the mice were randomly grouped prior to be treated. For other experiments, cells were planted randomly in wells prior to treatment.
Blinding	The investigators were not blinded to allocation during experiments and outcome assessment.

Reporting for specific materials, systems and methods

We require information from authors about some types of materials, experimental systems and methods used in many studies. Here, indicate whether each material, system or method listed is relevant to your study. If you are not sure if a list item applies to your research, read the appropriate section before selecting a response.

Materials & experimental systems

n/a	Involved in the study	
[ ]	[x]	Antibodies
[ ]	[x]	Eukaryotic cell lines
[x]	[ ]	Palaeontology and archaeology
[ ]	[x]	Animals and other organisms
[x]	[ ]	Clinical data
[x]	[ ]	Dual use research of concern
[x]	[ ]	Plants

Methods

n/a	Involved in the study	
[x]	[ ]	ChIP-seq
[ ]	[x]	Flow cytometry
[x]	[ ]	MRI-based neuroimaging

Antibodies

Antibodies used

anti-APOB antibody(proteintech, 20578-I-AP, Lot 00117266), anti-APOE antibody(proteintech, 66830-I-Ig, Lot 10008911), anti-HMGCR antibody(proteintech, 13533-1-AP, Lot 00120917) , anti-His antibody (proteintech, 10001-0-AP, Lot 00101471; 66005-1-Ig, Lot 10020245) anti-FLAG antibody, sigma, F1804-200µg, clone M2; anti-LDLR antibody, proteintech, 10785-1-AP, Lot 00118477; anti-LDLR antibody, R&D, AF2148, Lot VBC0221081; normal rabbit IgG, cell signaling technology, 2729p; ARH (LDLRAP1) Monoclonal antibody, proteintech, 66932-2-Ig, Lot 10008603; ARH (LDLRAP1) Monoclonal antibody, santa cruz, sc-514263, Lot #B2422; anti-SQLE antibody, santa cruz, sc-271651,Lot # A0421; anti-GAPDH antibody, santa cruz, sc-32233, Lot # I0319; APOE Monoclonal antibody, proteintech, 66830-1-Ig; LILRB1/CD85j (D4L8L) Rabbit mAb, cell signaling technology, 78144; Anti-LILRB1 antibody [EPR22861-6], abcam, ab238145, Lot: GR3416682-2; Recombinant Rabbit IgG, monoclonal [EPR25A] - Isotype Control, abcam, ab172730, Lot: CR3284310.13; anti-Mouse IgG, peroxidase-linked species-specific whole antibody (from sheep) Secondary Antibody, Cytiva, NA9311ML, Lot:17170538; anti-Rabbit IgG, peroxidase-linked species-specific whole antibody (from donkey) Secondary Antibody, Cytiva, NA9341ML, Lot 17271476; Donkey anti-Goat IgG (H+L) Highly Cross-Adsorbed Secondary Antibody, Alexa Fluor™ Plus 647, Invitrogen, A32849, Lot: WL333743; Donkey anti-Mouse IgG (H+L) Highly Cross-Adsorbed Secondary Antibody, Alexa Fluor™ Plus 555, Invitrogen, A32773, Lot: XC344355; Donkey anti-Rabbit IgG (H+L) Highly Cross-Adsorbed Secondary Antibody, Alexa Fluor™ 488, Invitrogen, A-21206, Lot:2376850; APC Annexin V, biolegend, 640941, Lot: B386061; APC anti-human CD85j (ILT2) Antibody, biolegend, 333720, clone GHI/75, Lot: B316130; APC Mouse IgG2b, κ Isotype Ctrl Antibody, biolegend, 400322, clone MPC-11, Lot: B317316; PE anti-human CD85j (ILT2) Antibody, biolegend, 333708, clone GHI/75, Lot: B274730; PE Mouse IgG2b, κ Isotype Ctrl Antibody, biolegend, 400314, clone: MPC-11; APC anti-human CD138 (Syndecan-1) Antibody, biolegend, 352308, clone: DL-101, Lot: B337398; PE anti-human CD138 (Syndecan-1) Antibody, biolegend, 352306, clone: DL-101, Lot: B259247; PE anti-Apo E Antibody, biolegend, 803405, clone: E6D7, Lot: B278267.

Validation

all validation can be accessed on the manufacturers' websites.

Eukaryotic cell lines

Policy information about cell lines and Sex and Gender in Research

Cell line source(s)

ARP-1 (CVCL_D523) cells were kindly provided by the Arkansas Cancer Research Center, Little Rock, AR. LP-1 (CVCL_0012), KMS-12-BM (CVCL_1334) and MWD MM.13 were kind gifts from Dr. Frederic J. Reu of the Cleveland Clinic63. MOLP-8 (CVCL_2124) cells were a kind gift from Dr. Jinsheng Weng, MD Anderson Cancer Center. ANBL-6 (Cat. # SCC429) was purchased from MilliporeSigma. Other cell lines (MM.1R,CRL-2975; NCI H929, CRL-9068; 8226, CCL-155; U266, TIB-196) were purchased from the American Type Culture Collection (Rockville, MD). All MM cells were cultured in 1% Pen/Strep, 10% FBS-containing RPMI-1640 medium (MilliporeSigma, R8758). Cells were tested routinely for mycoplasma and were free of contamination at the point of our experiments.

Authentication

STR were used for authentication of cell lines.

Mycoplasma contamination

all cell lines are tested negative for mycoplasma contamination.

Commonly misidentified lines
(See ICLAC register)

no commonly misidentified cell lines were used in the study.

Animals and other research organisms

Policy information about studies involving animals; ARRIVE guidelines recommended for reporting animal research, and Sex and Gender in Research

Laboratory animals

6-8 week immunodeficient NSG (Stock No:005557) mice were purchased from The Jackson Laboratory. All mice were kept under clean conditions in 12/12 light/dark cycle,65-75°F and 40-60% humidity.

Wild animals

The study did not involve wild animals.

Reporting on sex

For mouse experiments, we repeated our key experiment (the effect ofLILRB1 knockdown in vivo) with both female and male mice, and found that knockdown of LILRB1 on MM cells inhibit the progression in vivo in both femal and male mice. Therefore, in our system the results are similar between genders. For other mice experiments (overexpression of MM1R or liproxstatin-1) we use female mice only.

Field-collected samples	All mice were kept under clean conditions in 12/12 light/dark cycle,65-75°F and 40-60% humidity. We collect blood from the tail vein and 3-5 drops of blood were taken once a week. Discomfort, distress, pain of the mice in these experiments will be kept to a minimum. Tumor-bearing mice will be euthanized when mice develop hind-leg paralysis or when mice are in obvious distress or moribund.
Ethics oversight	All mouse studies were approved by the Institutional Animal Care and Use Committee of the Houston Methodist Research Institute.

Note that full information on the approval of the study protocol must also be provided in the manuscript.

Flow Cytometry

Plots

Confirm that:

- [x] The axis labels state the marker and fluorochrome used (e.g. CD4-FITC).
- [x] The axis scales are clearly visible. Include numbers along axes only for bottom left plot of group (a 'group' is an analysis of identical markers).
- [x] All plots are contour plots with outliers or pseudocolor plots.
- [x] A numerical value for number of cells or percentage (with statistics) is provided.

Methodology

Sample preparation	For detection of lipid ros, we used BODIPY™ 665/676 dye, which was purchased from Thermo Fisher Scientific. Briefly, cells were incubated in a humidified chamber at 37°C with 5% CO2 for 30 minutes with BODIPY™ 665/676 (Lipid Peroxidation Sensor) in cell culture medium. After incubation, cells were washed and examined by flow cytometry within 2 hours of staining. As BODIPY™ 665/676 exhibits a change in fluorescence after interaction with peroxyl radicals, the lipid ROS levels were presented by mean fluorescence (PE-CF594) divided by mean fluorescence (APC). The results were normalized to the untreated control cells. For detection of cell death: MM cells were harvested and washed with PBS buffer, followed by staining in PI solution (1:100 diluted in PBS buffer) for 15 minutes. PI positive cells were detected and analyzed to show the percentage of cell death by flow cytometry .
Instrument	BD FACS Symphony A3, BD Biosciences
Software	data was collected with the BD FACSDiva software and analyzed with the BD Flowjo software.
Cell population abundance	we gated all cells for analysis of cell death.
Gating strategy	Debris was excluded by a morphology gate based on FSC-A and SSC-A. Then cells were analysis.

- [x] Tick this box to confirm that a figure exemplifying the gating strategy is provided in the Supplementary Information.
